# Supplementary material for: Global endometrial DNA methylation analysis reveals insights into mQTL regulation and associated endometriosis disease risk and endometrial function
Source: Commun Biol. 2023 Aug 16;6:780. doi: 10.1038/s42003-023-05070-z (PMC10432557; doi:10.1038/s42003-023-05070-z)
Supplement: Supplementary file 3 — Description of Additional Supplementary Files [file 42003_2023_5070_MOESM3_ESM.pdf]

Description of Additional Supplementary Files

File name: Supplementary Data 1

Description: Summary of endometriosis case and control ascertainment criteria and subphenotypes for each methylation and genetic dataset contributing to the analysis.

File name: Supplementary Data 2

Description: Phenotypic variance captured by endometrial DNAm. Proportion of variance in endometriosis case-control status captured by common genetic variants and genome-wide DNA methylation (DNAm) in endometrium estimated using different GREML and OREML models. GRM is genetic relationship matrix and ORM is omic-relationship matrix.

File name: Supplementary Data 3

Description: Values of weighted partial R2 (%) from PC-PR2 analysis indicating the proportion of variability of methylation levels, before and after correction with surrogate variables, explained by covariates.

File name: Supplementary Data 4

Description: Significantly differentially methylated CpG sites between menstrual cycle phases.

File name: Supplementary Data 5

Description: Significantly differentially methylated regions between menstrual cycle phases.

File name: Supplementary Data 6

Description: Genomic locations of CpG Sites significantly associated with Cycle Phase.

File name: Supplementary Data 7

Description: Pathway analysis of genes annotated to differentially methylated CpG sites between menstrual cycle phases. N is the total number of genes in the pathway and DM is the number of genes from the pathway that were annotated to the differentially methylated CpG sites.

File name: Supplementary Data 8

Description: Pathway analysis of genes annotated to differentially methylated CpG sites in clusters associated with menstrual cycle phase from WGCNA. N is the total number of genes in the pathway and DM is the number of genes from the pathway that were annotated to the differentially methylated CpG sites.

File name: Supplementary Data 9

Description: WGCNA Pathway Analysis. Pathway analysis of genes annotated to CpG sites in modules associated with case:control. N is the total number of genes in the pathway and DM is the number of genes from the pathway that were annotated to the differentially methylated CpG sites.

File name: Supplementary Data 10

Description: Genomic Locations of CpG sites located in WGCNA modules.

File name: Supplementary Data 11

Description: Summary statistics for potential endometrial specific mQTLs.

File name: Supplementary Data 12

Description: Pathways enriched for genes annotated to potential endometrial specific mQTLs. N is the total number of genes in the pathway and DM is the number of genes from the pathway that were annotated to the differentially methylated CpG sites.

File name: Supplementary Data 13

Description: Context specific mQTLs for menstrual cycle stage and endometriosis.

File name: Supplementary Data 14

Description: Endometrial meQTLs significantly associated with overall endometriosis and stage III/IV endometriosis using SMR. Associated eQTLs are those significantly associated with the meQTL using SMR. Associated eQTLs highlighted in red are those significantly associated with endometriosis using SMR. Enhancer and promoter evidence for each tissue/cell type is based on predicted chromatin states using the 18-state Roadmap model from histone marks (H3K27ac, H3K4me1, H3K4me3, H3K36me3, H3K9me3, H3K27me3)(observed and imputed) using ChromHMM

File name: Supplementary Data 15

Description: SMR results for eQTLs significantly associated with SMR significant mQTLs.

File name: Supplementary Data 16

Description: SMR results for eQTLs significantly associated with endometriosis.

File name: Supplementary Data 17

Description: Pathways significantly enriched for genes annotated to predicted promotors and enhancers and eQTLs. N is the total number of genes in the pathway and DM is the number of genes from the pathway that were annotated to the differentially methylated CpG sites.

File name: Supplementary Data 18

Description: Summary and description of the endometriosis sub-phenotype comparisons.

File name: Supplementary Data 19

Description: Results of the 66 probes that were significantly differentially methylated in either of the 18 phenotypes including the effect-sizes, p-values from the differential methylation analysis, annotation of the probes and if any mQTLs in endometrium for these probes, the mQTL results and the LD with endometriosis GWAS loci.

File name: Supplementary Data 20

Description: Summary endometriosis cases and controls for each GWAS dataset contributing to the European GWAS meta-analysis.
