# Supplementary material for: Global endometrial DNA methylation analysis reveals insights into mQTL regulation and associated endometriosis disease risk and endometrial function
Source: Commun Biol. 2023 Aug 16;6:780. doi: 10.1038/s42003-023-05070-z (PMC10432557; doi:10.1038/s42003-023-05070-z)
Supplement: Supplementary file 5 — Reporting Summary [file 42003_2023_5070_MOESM5_ESM.pdf]

nature portfolio

Corresponding author(s): Sally Mortlock
Linda Giudice

Last updated by author(s): Apr 14, 2023

Reporting Summary

Nature Portfolio wishes to improve the reproducibility of the work that we publish. This form provides structure for consistency and transparency in reporting. For further information on Nature Portfolio policies, see our Editorial Policies and the Editorial Policy Checklist.

Statistics

For all statistical analyses, confirm that the following items are present in the figure legend, table legend, main text, or Methods section.

n/a	Confirmed	
☐	☒	The exact sample size (*n*) for each experimental group/condition, given as a discrete number and unit of measurement
☐	☒	A statement on whether measurements were taken from distinct samples or whether the same sample was measured repeatedly
☐	☒	The statistical test(s) used AND whether they are one- or two-sided<br>*Only common tests should be described solely by name; describe more complex techniques in the Methods section.*
☐	☒	A description of all covariates tested
☐	☒	A description of any assumptions or corrections, such as tests of normality and adjustment for multiple comparisons
☐	☒	A full description of the statistical parameters including central tendency (e.g. means) or other basic estimates (e.g. regression coefficient) AND variation (e.g. standard deviation) or associated estimates of uncertainty (e.g. confidence intervals)
☐	☒	For null hypothesis testing, the test statistic (e.g. *F*, *t*, *r*) with confidence intervals, effect sizes, degrees of freedom and *P* value noted<br>*Give P values as exact values whenever suitable.*
☒	☐	For Bayesian analysis, information on the choice of priors and Markov chain Monte Carlo settings
☐	☒	For hierarchical and complex designs, identification of the appropriate level for tests and full reporting of outcomes
☐	☒	Estimates of effect sizes (e.g. Cohen's *d*, Pearson's *r*), indicating how they were calculated

Our web collection on statistics for biologists contains articles on many of the points above.

Software and code

Policy information about availability of computer code

Data collection	no software was used to collect data
Data analysis	Several software programs were used to analyse data in this study (SeSAME R package, Minfi R package, SmartSVA R package, Limma R package, Complex Heatmap R package, Omic-data-based Complex Trait Analysis (OSCA), IlluminaHumanMethylationEPICanno.ilm10b4.hg19 annotation R package, clusterProfiler R package, enrichplot R package, DMRCate R package, Weighted correlation network analysis (WGCNA) , MatrixeQTL R package, GCTA, Summary-data-based Mendelian Randomization (SMR), PhenoScanner, GWAS Catalog, EpiMap) and all are described in the methods section. No custom code was used but code will be made available on GitHub before publication.

For manuscripts utilizing custom algorithms or software that are central to the research but not yet described in published literature, software must be made available to editors and reviewers. We strongly encourage code deposition in a community repository (e.g. GitHub). See the Nature Portfolio guidelines for submitting code & software for further information.

Data

Policy information about availability of data

All manuscripts must include a data availability statement. This statement should provide the following information, where applicable:

- Accession codes, unique identifiers, or web links for publicly available datasets
- A description of any restrictions on data availability
- For clinical datasets or third party data, please ensure that the statement adheres to our policy

Methylation data used in this study has been deposited and is available from GEO (GEO: GSE223817;Reviewer access using token: odkbqgaknzctpql). Genotype data generated in this study is available upon approval from dbGAP (accession number on acceptance). Code used to run the analyses is available on github (link). Any additional information required to reanalyze the data reported in this paper is available from the lead contact upon request.

Human research participants

Policy information about studies involving human research participants and Sex and Gender in Research.

Reporting on sex and gender: Only females were included in this study as it examined a female specific tissue and disorder.

Population characteristics: The following woman-level covariates were included in statistical modeling. Site: A categorical variable with a unique value for each of the five sample contributing sites/institutions (UCSF, ENDOX, UM, EDIN). Cycle phase: A categorical variable with a unique value for each of the six menstrual cycle phases (Menstrual, PE, SE, ESE, MSE, LSE). Endometriosis case:control status: A binary variable assigning samples as either a case or control. Sample plate: A categorical variable with a unique value for each of the 12 sample plates used during processing. Batch: A binary variable assigning samples as either Batch I or II.

Endometriosis sub-phenotyping characteristics included: rASRM endometriosis disease stage: visualized at surgery most proximal to endometrial biopsy collection and defined by the rASRM endometriosis scoring system. Stage data were used to create variables with three structures – continuous rASRM score, ordinal stages I, II, III, IV, and dichotomized as stage I+II and stage III+IV. For case patients for whom surgical documentation was noted as the rASRM stage category only, the variables were categorized as documented. However, four patients were defined in their surgical record as having visualized stage II-III and were assigned to the I-II dichotomized rASRM category. Lesion type: categorized according to the presence of at least one superficial peritoneal lesion, endometrioma, or deep lesion. Lesion types were binary variables coded as “any” peritoneal lesion, endometrioma, or deep lesion, regardless of co-occurrence of another lesion type and not mutually exclusive variables. Pain: binary variables for the presence or absence of dyspareunia, acyclic pelvic pain and dyschezia.

Recruitment: Endometrial tissue from 679 surgically-diagnosed endometriosis patients (cases), 389 controls without endometriosis and six participants with unconfirmed endometriosis status were recruited through the University of California San Francisco, California (UCSF, n=480 samples), University of Melbourne, Melbourne, Australia (UM, n=315 samples), Endometriosis CaRe Centre in Oxford, Oxford, UK (ENDOX, n=193 samples), and EXPPECT Centre, The University of Edinburgh (EDIN), Edinburgh, Scotland, UK (n=86 samples), with collection at all sites using the World Endometriosis Research Foundation Endometriosis Phenome and Biobanking Harmonization Project (WERF EPHect) standardized protocols for tissue collection and processing, and participant characteristics and clinical annotation.

Ethics oversight: All participants provided the site-specific study investigator with informed consent. All patient data were de-identified and followed HIPAA and the Convention of the Declaration of Helsinki. This study was approved by the institutional review boards of UCSF (Administrative Multi-Principal Investigator site), Michigan State University (Multi-Principal Investigator site), University of Oxford, University of Melbourne and University of Edinburgh.

Note that full information on the approval of the study protocol must also be provided in the manuscript.

Field-specific reporting

Please select the one below that is the best fit for your research. If you are not sure, read the appropriate sections before making your selection.

☒ Life sciences ☐ Behavioural & social sciences ☐ Ecological, evolutionary & environmental sciences

For a reference copy of the document with all sections, see nature.com/documents/nr-reporting-summary-flat.pdf

Life sciences study design

All studies must disclose on these points even when the disclosure is negative.

Sample size: 1,074 samples collected for this study

Data exclusions: Participants were restricted to those who had not been on contraceptive steroids or gonadotropin releasing hormone analogues for 3 months or more prior to endometrial sampling, had regular cycles (defined as 24-35 days in length) and no evidence of endometrial hyperplasia or

cancer. Samples were required to pass all quality control (QC) metrics [high DNA integrity (ALU CT<25), 100% bisulfite conversion with no amplifications at 0% or 50% conversion]. Unsuitable samples (inactive, atrophic, PE/SE, progestin effect, dyssynchronous) were excluded. “Benign” histology descriptor and “unknown” were assigned as “unknown” in the absence of last menstrual period (LMP) and/or serum E2 and P4 levels (assayed at University of Virginia NIH National Institute for Child Health and Human Development (NICHD) Ligand Core). Endometrial samples from women with a history of endometriosis but no disease identified at surgery were not considered as controls and were not suitable for DNAm quantification, and thus were excluded from that analysis. The following filtering steps for samples were carried out. Samples with a low overall intensity signal, defined as a median unmethylated or methylated signal <9, were removed from the dataset. In addition to this, samples were also filtered out if they had a detection p-value >0.05 in more than 1% of DNAm sites. Samples with genotype call rate < 95%, heterozygosity rate > 3 standard deviations away from the mean heterozygosity rate and related (IBD > 0.200) samples were removed from genetic analyses.

Replication	This iss the largest dataset in endometrium and was able to replicate some findings from smaller studies whilst reporting novel findings.
Randomization	Experimental groups were assigned based on disease and menstrual phase phenotypes for the purpose of analysis. For molecular assays samples were randomly run on array chips.
Blinding	Blinding was not relevant

Reporting for specific materials, systems and methods

We require information from authors about some types of materials, experimental systems and methods used in many studies. Here, indicate whether each material, system or method listed is relevant to your study. If you are not sure if a list item applies to your research, read the appropriate section before selecting a response.

Materials & experimental systems

n/a	Involved in the study	
☒	☐	Antibodies
☒	☐	Eukaryotic cell lines
☒	☐	Palaeontology and archaeology
☒	☐	Animals and other organisms
☒	☐	Clinical data
☒	☐	Dual use research of concern

Methods

n/a	Involved in the study	
☒	☐	ChIP-seq
☒	☐	Flow cytometry
☒	☐	MRI-based neuroimaging
